# High-Mobility Group Box 1 Inhibits Gastric Ulcer Healing through Toll-Like Receptor 4 and Receptor for Advanced Glycation End Products

**DOI:** 10.1371/journal.pone.0080130

**Published:** 2013-11-11

**Authors:** Yuji Nadatani, Toshio Watanabe, Tetsuya Tanigawa, Fumikazu Ohkawa, Shogo Takeda, Akira Higashimori, Mitsue Sogawa, Hirokazu Yamagami, Masatsugu Shiba, Kenji Watanabe, Kazunari Tominaga, Yasuhiro Fujiwara, Koji Takeuchi, Tetsuo Arakawa

**Affiliations:** 1 Department of Gastroenterology, Osaka City University Graduate School of Medicine, Osaka, Japan; 2 Division of Pathological Sciences, Department of Pharmacology and Experimental Therapeutics, Kyoto Pharmaceutical University, Kyoto, Japan; INSERM, France

## Abstract

High-mobility group box 1 (HMGB1) was initially discovered as a nuclear protein that interacts with DNA as a chromatin-associated non-histone protein to stabilize nucleosomes and to regulate the transcription of many genes in the nucleus. Once leaked or actively secreted into the extracellular environment, HMGB1 activates inflammatory pathways by stimulating multiple receptors, including Toll-like receptor (TLR) 2, TLR4, and receptor for advanced glycation end products (RAGE), leading to tissue injury. Although HMGB1’s ability to induce inflammation has been well documented, no studies have examined the role of HMGB1 in wound healing in the gastrointestinal field. The aim of this study was to evaluate the role of HMGB1 and its receptors in the healing of gastric ulcers. We also investigated which receptor among TLR2, TLR4, or RAGE mediates HMGB1’s effects on ulcer healing. Gastric ulcers were induced by serosal application of acetic acid in mice, and gastric tissues were processed for further evaluation. The induction of ulcer increased the immunohistochemical staining of cytoplasmic HMGB1 and elevated serum HMGB1 levels. Ulcer size, myeloperoxidase (MPO) activity, and the expression of tumor necrosis factor α (TNFα) mRNA peaked on day 4. Intraperitoneal administration of HMGB1 delayed ulcer healing and elevated MPO activity and TNFα expression. In contrast, administration of anti-HMGB1 antibody promoted ulcer healing and reduced MPO activity and TNFα expression. TLR4 and RAGE deficiency enhanced ulcer healing and reduced the level of TNFα, whereas ulcer healing in TLR2 knockout (KO) mice was similar to that in wild-type mice. In TLR4 KO and RAGE KO mice, exogenous HMGB1 did not affect ulcer healing and TNFα expression. Thus, we showed that HMGB1 is a complicating factor in the gastric ulcer healing process, which acts through TLR4 and RAGE to induce excessive inflammatory responses.

## Introduction

High-mobility group box protein 1 (HMGB1), a member of the high-mobility group protein superfamily, is a nuclear protein [1]. HMGB1 interacts with DNA as a chromatin-associated nonhistone protein to stabilize nucleosomes and to regulate the transcription of many genes in the nucleus [2]. When leaked from a cell during necrotic cell death [3] or actively secreted into the extracellular environment by monocytes and macrophages [3,4], HMGB1 acts as an alarmin with potent proinflammatory properties [5]. 

The best studied HMGB1 receptors are Toll-like receptor (TLR) 2 [6,7], TLR 4 [6-9], and receptor for advanced glycation end products (RAGE) [6,8]. TLR2 and TLR4 are members of the TLR family, and they play a crucial role in innate immune responses to pathogen-associated molecular patterns and damage-associated molecular pattern molecules [10]. TLR2 primarily recognizes components of the gram-positive bacterial cell wall, and TLR4 primarily recognizes lipopolysaccharide, which is the major cell wall component of gram-negative bacteria. Triggering TLR2 and TLR4 signaling pathways leads to the activation of nuclear factor κB (NF-κB), through the accessory protein MyD88, and the subsequent regulation of immune and inflammatory genes, including inflammatory cytokines such as tumor necrosis factor α (TNFα), with the activation of mitogen-activated protein kinases [11-13]. Receptor for advanced glycation end products (RAGE) is a multi-ligand receptor that belongs to the immunoglobulin superfamily [14]. Other known RAGE ligands include amyloid [15] and S100 [16]. Multiple experiments have suggested that the ligand-RAGE interaction also activates NF-κB and mitogen-activated protein kinases [17-20]. 

Many pathological conditions are related to the proinflammatory properties of HMGB1. Previous reports demonstrated that HMGB1 plays a critical role in endotoxemia [21], acute pancreatitis [22], acute respiratory distress syndrome [23], some autoimmune diseases [24], cerebral ischemia injury [25], and ischemia-reperfusion (I-R) injuries of the liver [26], heart [27], and kidney [28]. With regard to the gastrointestinal tract, HMGB1 is a complicating factor in experimental colitis [29,30], and non-steroidal anti-inflammatory drug induced small intestinal injury [31]. 

At present, the role of HMGB1 in wound healing is unclear, although its ability to induce inflammation has been well documented, as described above. In the gastrointestinal field, no study has examined the role of HMGB1 in wound healing. The aim of this study was to investigate the role of HMGB1 in gastric ulcer healing. We investigated the role of HMGB1 in the healing process by using an established experimental chronic gastric ulcer model created in rodent by topical application of acetic acid from the gastric serosal side. The model closely mimics human peptic gastric ulcer in histology and morphology [32]. We also investigated whether HMGB1 affects ulcer healing through TLR2, TLR4, or RAGE. 

## Materials and Methods

### Animals

TLR2- and TLR4-knockout (KO) mice, which were originally generated by Dr. S. Akira (Osaka University, Osaka, Japan) and backcrossed 8 times onto a C57BL/6 background, were obtained from Oriental Bioservice, Inc. (Kyoto, Japan). RAGE-KO mice, which had been backcrossed onto a C57BL/6 background, were originally generated by and a gift from Dr. Y. Yamamoto (Kanazawa Medical University, Kanazawa, Japan). Wild-type C57BL/6 mice were purchased from Charles River Japan, Inc. (Atsugi, Japan) as the control strain for TLR2 KO, TLR4 KO, and RAGE KO mice. Specific pathogen-free 12-week-old male animals were used. All animals were housed in polycarbonate cages with paper chip bedding. The cages were located in an air-conditioned biohazard room with a 12-h light-dark cycle. All experimental procedures were approved by the Animal Care Committee of the Osaka City University Graduate School of Medicine (Permit Number: 11006). All surgeries were performed under isoflurane, and all efforts were made to minimize suffering.

### Experimental Induction of Ulcer

Gastric ulcer was induced by a method described in detail elsewhere [33], with minor modifications. Briefly, under ether anesthesia, the abdomens of the animals were incised and the stomach was exposed. A polypropylene tube (4 mm in diameter) was placed against the serosal side of the stomach. An 80-μL aliquot of 60% acetic acid was added to the tube, which was kept in contact with the serosal surface for 30 s. After immediate removal of acetic acid from the tube by aspiration, the stomach was returned to its original position, and the abdomen was closed. Previous reports demonstrated that the size of the gastric ulcer reached a maximum on day 3 or 4 after ulcer induction, and thereafter, it gradually decreased [32,34]. The healing phase of the experimental gastric ulcer starts on day 4 after ulcer induction.

### Experimental Groups

To investigate the effect of exogenous HMGB1, mice received intraperitoneal injections of human recombinant HMGB1 (rHMGB1; 100–1000 μg/kg; Sigma-Aldrich Co., St. Louis, MO) or vehicle (phosphate-buffered saline) twice daily, beginning at 4 days after ulcer induction (from day 4 to day 9). 

Next, the effect of immunoneutralization of HMGB1 on gastric ulcer healing was assessed. Mice were intraperitoneally administered neutralizing chicken anti-HMGB1 polyclonal antibody (5 mg/kg; Shino-Test Corporation, Tokyo, Japan) or normal chicken IgY (5 mg/kg; Sigma-Aldrich Co.) beginning at 4 days after ulcer induction (from day 4 to day 9). Moreover, to confirm the effect of release of an inhibitor of HMGB1, ethyl pyruvate or vehicle were injected twice daily, beginning on day 4 after ulcer induction.

Furthermore, to determine the receptor responsible for HMGB1-related gastric ulcer healing, gastric ulcers were induced in TLR2 KO, TLR4 KO, and RAGE KO mice with or without intraperitoneal injection of 1000 μg/kg of rHMGB1 twice daily beginning on day 4 after ulcer induction. 

The stomach was removed and the ulcer size was measured on day 6 or 9 after ulcer induction. Ulcer size was expressed as an ulcer index, the product of the maximum length and minimum length (i.e. maximum length was multiplied by minimum length). Studies were carried out using 4–8 samples. The samples of gastric tissue were processed for further evaluation.

### mRNA Expression of Inflammatory Mediators in Gastric Tissue Determined by Real-time Quantitative Reverse Transcription-Polymerase Chain Reaction (RT-PCR)

Real-time quantitative RT-PCR was performed as previously described [35]. In brief, total RNA was isolated from intestinal tissue by using an ISOGEN kit (Nippon Gene Co., Ltd., Tokyo, Japan) according to the manufacturer’s protocol. Complementary DNA was acquired using a High Capacity RNA-to-cDNA Kit (Life Technologies Corporation, Carlsbad, CA) according to the manufacturer’s protocol. Real-time quantitative RT-PCR analyses were performed using an Applied Biosystems 7500 Fast Real-Time PCR system and software (Life Technologies Corporation). The reaction mixture was prepared according to the manufacturer’s protocol by using the TaqMan Fast Universal PCR master mixture (Life Technologies Corporation). Thermal cycling conditions were as follows: 45 cycles of amplification at 95°C for 15 s and 60°C for 1 min. Total RNA was subjected to real-time quantitative RT-PCR for the measurement of target genes using TaqMan glyceraldehyde-3-phosphate dehydrogenase control reagents (Life Technologies Corporation), which were used as an internal standard. The expression of mRNA encoding HMGB1, TLR4, RAGE, vascular endothelial growth factor (VEGF), interleukin-1-beta (IL-1β), and TNFα in ulcerated and normal gastric tissues was quantified using real time RT-PCR and standardized to glyceraldehyde-3-phosphate dehydrogenase mRNA levels. The expression of each mRNA is indicated as a ratio, relative to the mean value in normal gastric tissue. The primers and probes used for RT-PCR are shown in Table 1.

**Table 1:** Primers and Probes.

Gene		Primer and Probe
TNF-α	Primer (forward)	5’-TCATGCACCACCATCAAGGA-3’
	Primer (reverse)	5’-GAGGCAACCTGACCACTCTCC-3’
	Probe	5’-FAM-AATGGGCTTTCCGAATTCACTGGAGC-TAMRA-3’
IL-1β	Primer (forward)	5’-ACAGGCTCCGAGATGAACAAC-3’
	Primer (reverse)	5’-CCATTGAGGTGGAGAGCTTTC-3’
	Probe	5’-FAM-GAAAAAGCCTCGTGCTGTCGGACCCATAT-TAMRA-3’
RAGE	Primer (forward)	5’-CCACTGGATAAAGGATGGTGCA-3’
	Primer (reverse)	5’-CAGCTATAGGTGCCCTCATCCTC-3’
	Probe	5’-FAM-AGCCCTGTGCTGCTCCTCCCTGAG-TAMRA-3’
TLR2	Primer (forward)	5’-CTCTGGAGCATCCGAATTGC-3’
	Primer (reverse)	5’-GCTGAAGAGGACTGTTATGGC-3’
	Probe	5’-CCTCAGACAAAGCGTCAAATCTCAGAGGA-TAMRA-3’
TLR4	Primer (forward)	5’-GGCTGGATTTATCCAGGTGTGA-3’
	Primer (reverse)	5’-CTGTCAGTATCAAGTTTGAGAGGTG-3’
	Probe	5’-AGCCATGCCATGCCTTGTCTTCAATTGT-TAMRA-3’
HMGB1	Primer (forward)	5’-CAGCCATTGCAGTACATTGAGC-3’
	Primer (reverse)	5’-TCTCCTTTGCCCATGTTTAGTTG-3’
	Probe	5’-GACAGAGTCGCCCAGTGCCCGTCC-TAMRA-3’
VEGF	Primer (forward)	5’-TCCGCAGACGTGTAAATGTTC-3’
	Primer (reverse)	5’-TTAACTCAAGCTGCCTCGCCT-3’
	Probe	5’-FAM-TGCAAAAACACACAGACTCGCGTTGC-TAMRA-3’

### Immunohistochemical and Immunofluorescence Staining

Tissue samples were fixed with 0.1 M phosphate buffer (pH 7.4) containing 4% paraformaldehyde. Samples were embedded in paraffin, and serial 5-μm-thick sections were mounted on silanized slides (Dako, Tokyo, Japan). The specimens were immersed in a solution of 3% H_2_O_2_ in absolute methanol for 5 min in order to inhibit endogenous peroxidase activity and then incubated in 5% skim milk for 10 min. Hematoxylin and eosin staining was performed for the morphological observations. A rabbit monoclonal anti-HMGB1 antibody (diluted 1:250, Abcam, Cambridge, MA) was applied as the primary antibody and incubated overnight at 4°C with the specimens. A Secondary antibody (Histofine Simple Stain MAX Peroxidase kit; Nichirei Biosciences Inc., Tokyo, Japan) was incubated with the specimens for 1 h according to the manufacturer’s instructions. Immunoreactivity was visualized by treating the sections with Histofine Simple Stain and diaminobenzidine solution (Nichirei Biosciences Inc.). The specimens were then counterstained with hematoxylin. Next, TLR2, TLR4 and RAGE expression was determined by an immunofluorescence method. The primary antibodies used in immunofluorescence staining included a mouse monoclonal antibody against TLR2 (diluted 1:200; Abcam), a mouse monoclonal antibody against TLR4 (diluted 1:200; Abcam), and a rat monoclonal antibody against RAGE (diluted 1:250; Abcam). Tissue samples, which were prepared as described above, were incubated overnight at 4°C with the primary antibodies and then reacted with the corresponding fluorescent dye-conjugated secondary antibodies (Abcam) for 2 h. Samples were examined with a confocal microscope equipped with argon and argon-krypton laser sources. 

### Measurement of Serum HMGB1 Levels

Blood (1000 μL) samples were obtained in serum separator tubes by cardiac puncture. After centrifugation at 3,000 rpm for 10 min, the serum was collected and stored at −80°C. Serum levels of HMGB1 were measured using an HMGB1 sandwich ELISA kit (Shino-Test Corporation) according to the manufacturer’s protocol. 

### Measurement of Myeloperoxidase (MPO) Activity

Methods used to measure MPO activity are described in detail elsewhere [36]. In brief, the specimens were homogenized in 50 mM potassium phosphate buffer (pH 6.0) containing 0.5% hexadecyltrimethylammonium bromide (Sigma Chemical Co.). Suspensions were centrifuged, and MPO activity in the resulting supernatant was assayed with a spectrophotometer. One unit of MPO activity was defined as the amount of enzyme that degraded 1 μmol peroxide/min at 25°C. The results are expressed as units per gram of gastric tissue. 

### Statistical Analysis

Values are expressed as the mean ± standard error of the mean (SEM). One-way analysis of variance (ANOVA) was used to test the significance of the differences between treatment group means, and the results were analyzed with Fisher’s protected least-significant-difference test. P-values less than 0.05 were considered statistically significant. 

## Results

### Time Course of Gastric Ulcer Healing

**Figure 1:**
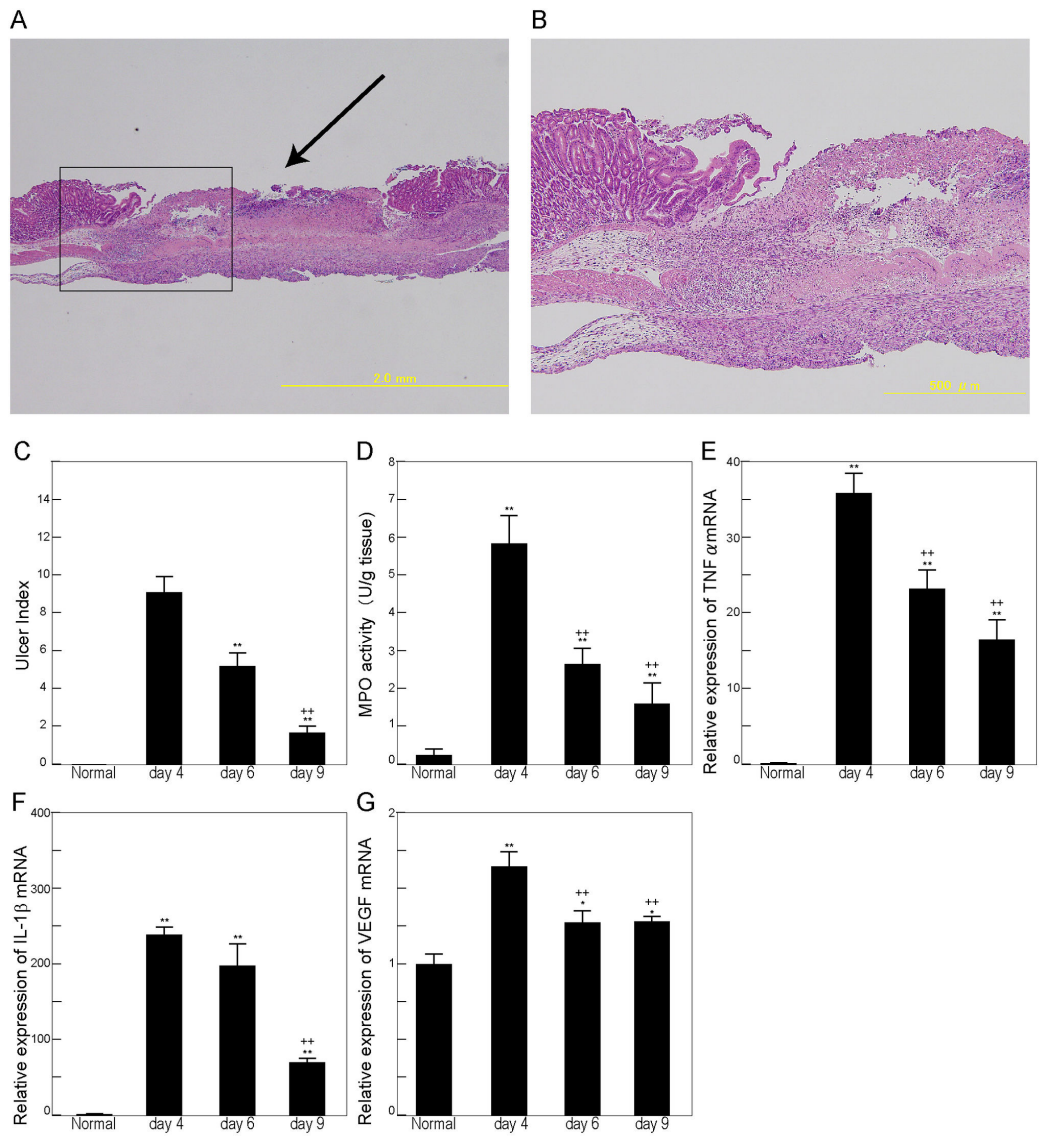
Ulcer index and cytokine expression following ulceration. A, B: Hematoxylin-eosin staining of gastric ulcer (day 6). The arrow indicates the ulcer site. B: Higher magnification of Figure 1A. C: Time course of the ulcer index following ulceration. The size of the macroscopically visible ulcer was expressed as an ulcer index, the product of maximum length and minimum length. D: Myeloperoxidase (MPO) activity of gastric tissue. One unit of MPO activity was defined as the amount of enzyme that degrades 1 μmol peroxide/min at 25°C. The results are expressed as units per gram of gastric tissue. E–H: The mRNA expression of tumor necrosis factor α (TNFα) (E), interleukin-1β (IL1-β) (F), and vascular endothelial growth factor (VEGF) (G) were determined by quantitative reverse transcription-polymerase chain reaction (RT-PCR). mRNA levels are expressed as ratios, relative to the mean value for normal gastric tissue. Each column represents the mean ± standard error of the mean ± SEM. N = 6–9. **P < 0.01, *P < 0.05 vs. untreated controls. ^++^P < 0.01, ^+^P < 0.05 vs. day 4 group.

To evaluate the healing process of gastric ulcers, experimental gastric ulcer was induced by topical application of acetic acid from the gastric serosal side. The ulcers were evaluated microscopically (Figure 1A, 1B) and macroscopically. The size of the ulcers reached a maximum on day 4 and decreased over time thereafter (Figure 1C). MPO activity (Figure 1D) and the expression of TNFα (Figure 1E) and IL-1β (Figure 1E) mRNA in the ulcerated gastric tissue also peaked on day 4, and their levels were higher in ulcerated tissue than in normal gastric tissue throughout the examination period. Ulceration also elevated the expression of VEGF mRNA (Figure 1F). 

### HMGB1 Expression following Ulceration

**Figure 2:**
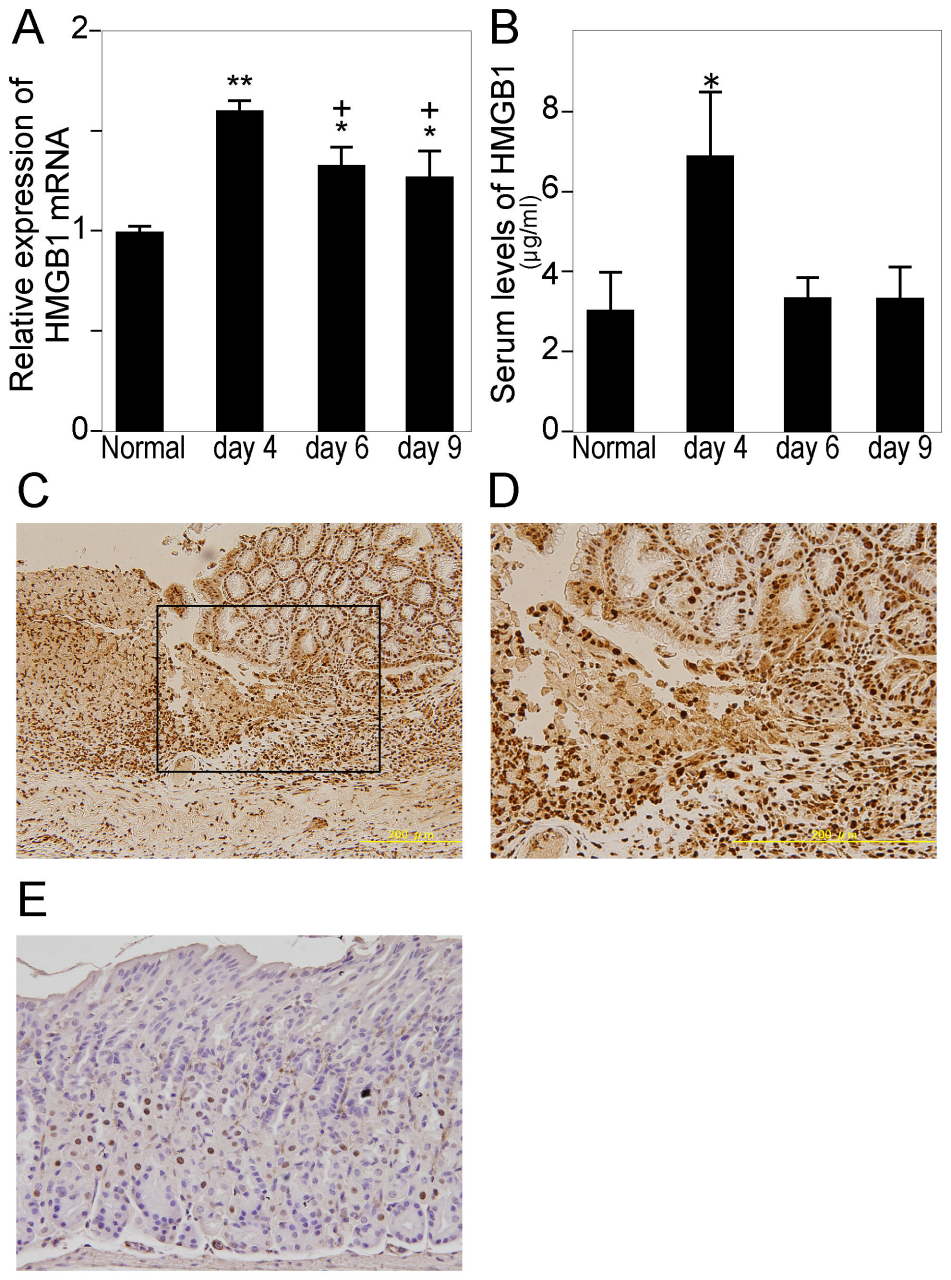
High mobility group box 1 (HMGB1) expression following ulceration. Changes in HMGB1 mRNA expression (A) and HMGB1 serum levels (B) during gastric ulcer healing. The levels of HMGB1 mRNA were determined by quantitative RT-PCR, and HMGB1 serum levels were determined by ELISA. HMGB1 mRNA levels are expressed as ratios, relative to the mean value of the gastric tissue in the control group (0-h group). Each column represents the mean ± SEM. N = 3–7. **P < 0.01, *P < 0.05 vs. control group. C, D: Immunohistochemistry of HMGB1 in ulcerated gastric tissue (day 6). HMGB1 localized to the cytoplasm in injured epithelial cells as well as to the nuclei of epithelial cells and interstitial cells. D: Higher magnification of Figure 2C. E: Immunohistochemistry of HMGB1 in untreated gastric tissue. HMGB1 localization was limited to the inside of nuclei of epithelial cells in intact gastric mucosa.

We next evaluated whether HMGB1 was involved in gastric ulcer healing. HMGB1 mRNA levels in gastric tissue (Figure 2A) and serum levels of HMGB1 (Figure 2B) reached their maximum at day 4. HMGB1 mRNA levels were almost constant during the examination period, whereas the serum levels of HMGB1 dropped to normal levels 6 days after the induction of ulcer. Immunohistochemically, ulceration induced prominent cytoplasmic staining of HMGB1 in epithelial cells, especially in injured areas (Figure 2C, 2D). In contrast, in intact gastric mucosa, HMGB1 localization was limited to inside the nuclei of epithelial cells (Figure 2E). 

### Effects of Exogenous HMGB1, HMGB1 Immunoneutralization, and Inhibition of HMGB1 Release on Gastric Ulcer Healing

**Figure 3:**
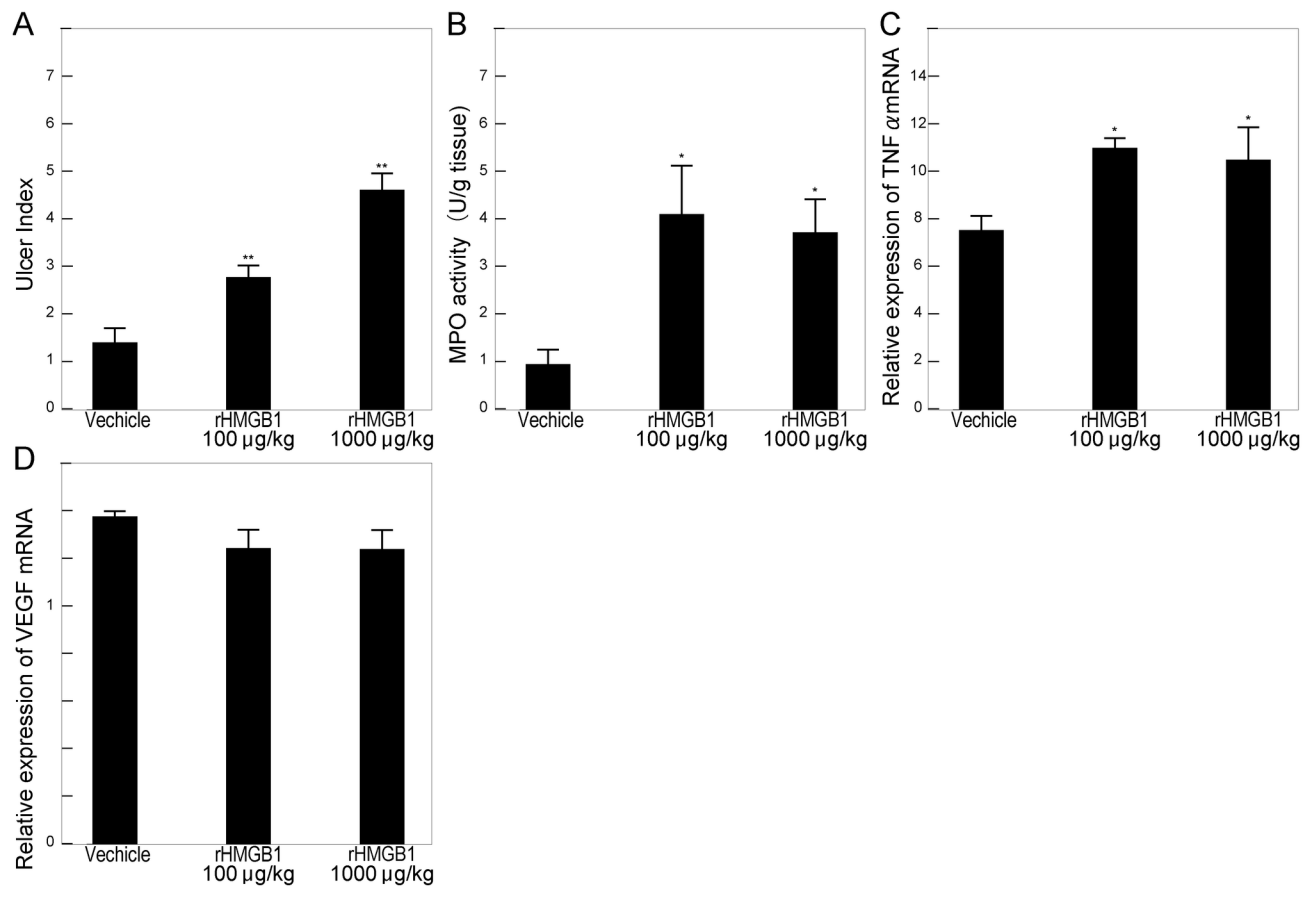
Effect of exogenous HMGB1 on the healing of gastric ulcers. Mice received intraperitoneal injections of human rHMGB1 (100–1000 μg/kg) or vehicle after ulceration. Ulcer index, mRNA expression, and myeloperoxidase (MPO) activity were measured in gastric tissues. Effects of exogenous HMGB1 on the ulcer index (A), MPO activity (B), and cytokine expression (C, D) following ulceration. Ulcer size was expressed as an ulcer index, the product of maximum length and minimum length. MPO activities (B) were measured according to Bradley’s methods, and the expression of TNFα (C) and VEGF (D) was determined by quantitative RT-PCR. Each column represents the mean ± SEM. N = 5–8. **P < 0.01, *P < 0.05 vs. vehicle-treated control group.

**Figure 4:**
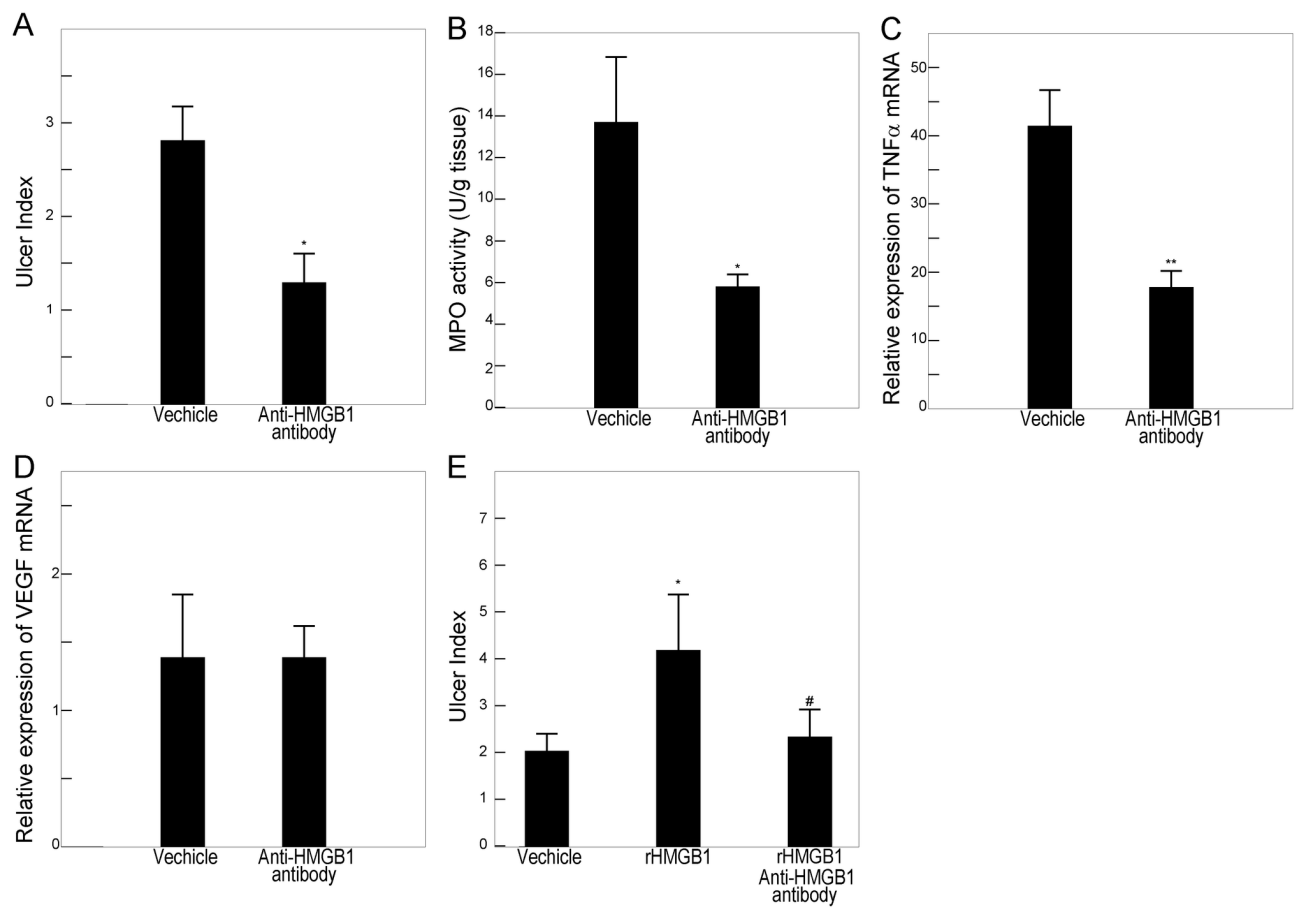
Effect of HMGB1 immunoneutralization on the healing of gastric ulcers. Mice received intraperitoneal injections of neutralizing chicken anti-HMGB1 polyclonal antibody (50 mg/kg) or vehicle after ulceration. Ulcer index, mRNA expression, and myeloperoxidase (MPO) activity were measured in gastric tissues. Effects of the anti-HMGB1 antibody on the ulcer index (A), MPO activity (B), and cytokine expression (C, D) following ulceration. Ulcer size was expressed as an ulcer index, the product of maximum length and minimum length. MPO activities (B) were measured according to Bradley’s methods, and the expression of TNFα (C) and VEGF (D) mRNA was determined by quantitative RT-PCR. Each column represents the mean ± SEM. N = 5–8. **P < 0.01, *P < 0.05 vs. vehicle-treated control group.

mRNA expression and MPO activity were evaluated on day 6, and the ulcer index was evaluated on day 9, according to the time course study. Mice were administered rHMGB1 or anti-HMGB1 antibody intraperitoneally following the induction of ulcer. Administration of rHMGB1 at a dose of either 100 μg/kg or 1000 μg/kg significantly suppressed gastric ulcer healing (Figure 3A), which was associated with increased MPO activity (Figure 3B) and TNFα mRNA expression in ulcerated tissues (Figure 3C). In contrast, HMGB1 neutralizing antibodies promoted ulcer healing (Figure 4A) with reduced MPO activity (Figure 4B) and TNFα mRNA expression (Figure 4C). The complicating effect of exogenous rHMGB1 on gastric ulcer healing was canceled by coadministration of anti-HMGB1 antibody (Figure 4F). Expression of VEGF mRNA was not affected by treatment with either rHMGB1 (Figure 3D, 3E) or anti-HMGB1 antibody (Figure 4D, 4E). In normal gastric tissue, administration of rHMGB1 or anti-HMGB1 also did not have any effects on cytokine expression or MPO activity (data not shown). Furthermore, the administration of ethyl pyruvate, an inhibitor of HMGB1 release, markedly promoted ulcer healing, compared with vehicle treatment. Healing was accompanied by the suppression of TNFα mRNA expression (data not shown).

### Role of TLR2, TLR4, and RAGE in Gastric Ulcer Healing

**Figure 5:**
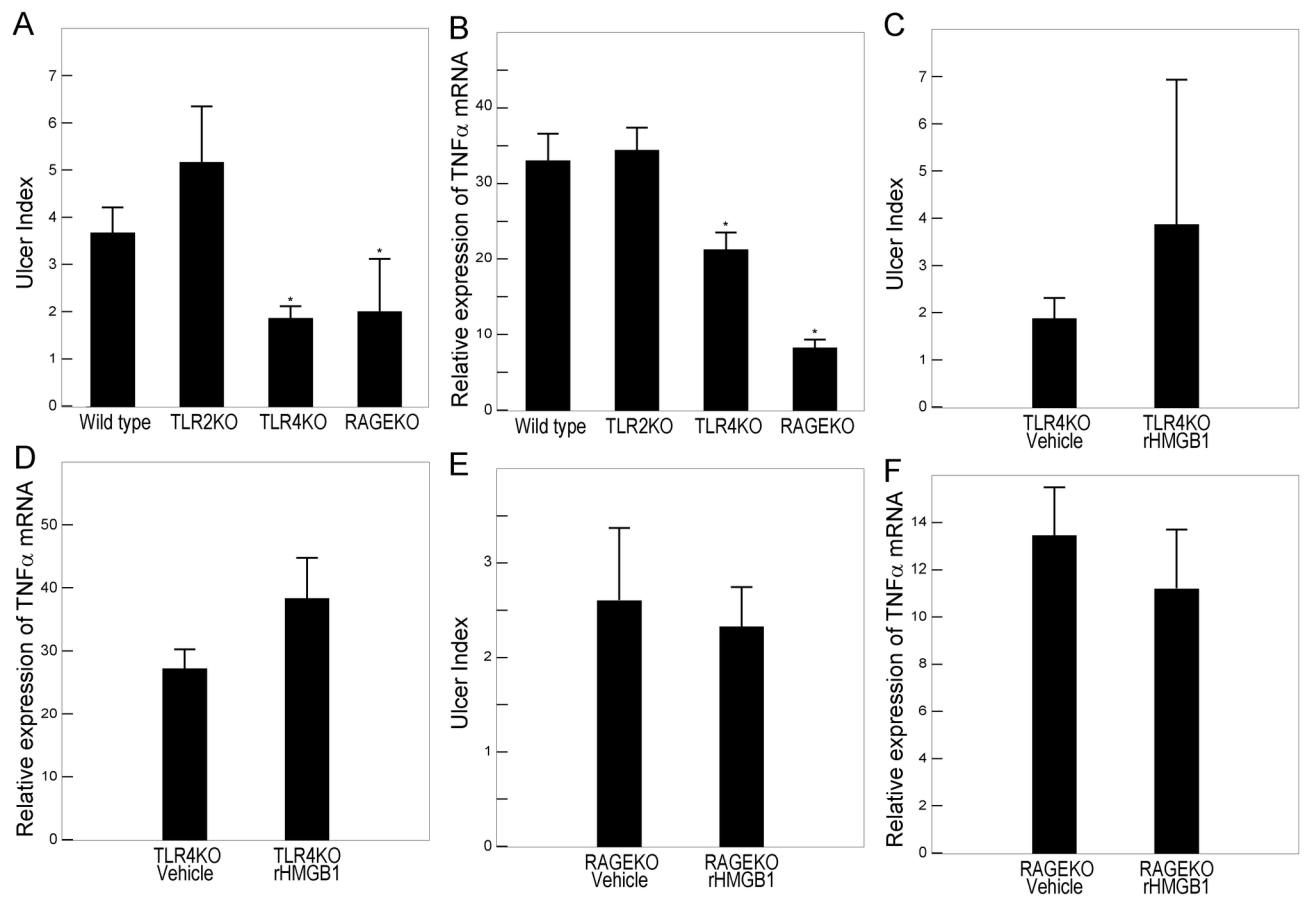
Role of TLR2, TLR4, and RAGE in gastric ulcer healing. Gastric ulcers were induced in TLR2 KO, TLR4 KO, and RAGE KO mice. The ulcer index (A) and TNFα mRNA levels (B) were measured in gastric tissue. C–F: Effect of recombinant HMGB1 on gastric ulcer healing in TLR4 KO and RAGE KO mice. Gastric ulcers were induced in TLR4 KO (C, D) and RAGE KO (E, F) mice with or without intraperitoneal injections of 1000 μg/kg rHMGB1. The ulcer index (A, C, E) and TNFα mRNA levels (B, D, F) were measured in gastric tissues. The mRNA levels assessed by RT-PCR are expressed as ratios, relative to the mean value for normal gastric tissue. Each column represents the mean ± SEM. N = 4–6. **P < 0.01, *P < 0.05 vs. wild-type mice.

To investigate whether TLR2, TLR4, and RAGE contribute to HMGB1-mediated gastric ulcer healing, experimental gastric ulcers were induced in TLR2 KO, TLR4 KO, RAGE KO, and control wild-type mice. TLR4 and RAGE deficiency promoted gastric ulcer formation and prevented the increase in TNFα mRNA expression after ulceration, whereas TLR2 deficiency affected neither the ulcer index nor the expression of TNFα mRNA (Figure 5A, 5B). Administration of exogenous HMGB1 affected neither the ulcer index nor the expression of TNFα mRNA in either TLR4 KO or RAGE KO mice (Figure 5C–5F). Administration of exogenous HMGB1, however, delayed ulcer healing in wild-type mice and reduced TNFα mRNA expression (Figure 3). 

### Expression of TLR2, TLR4 and RAGE during Gastric Ulcer Healing

**Figure 6:**
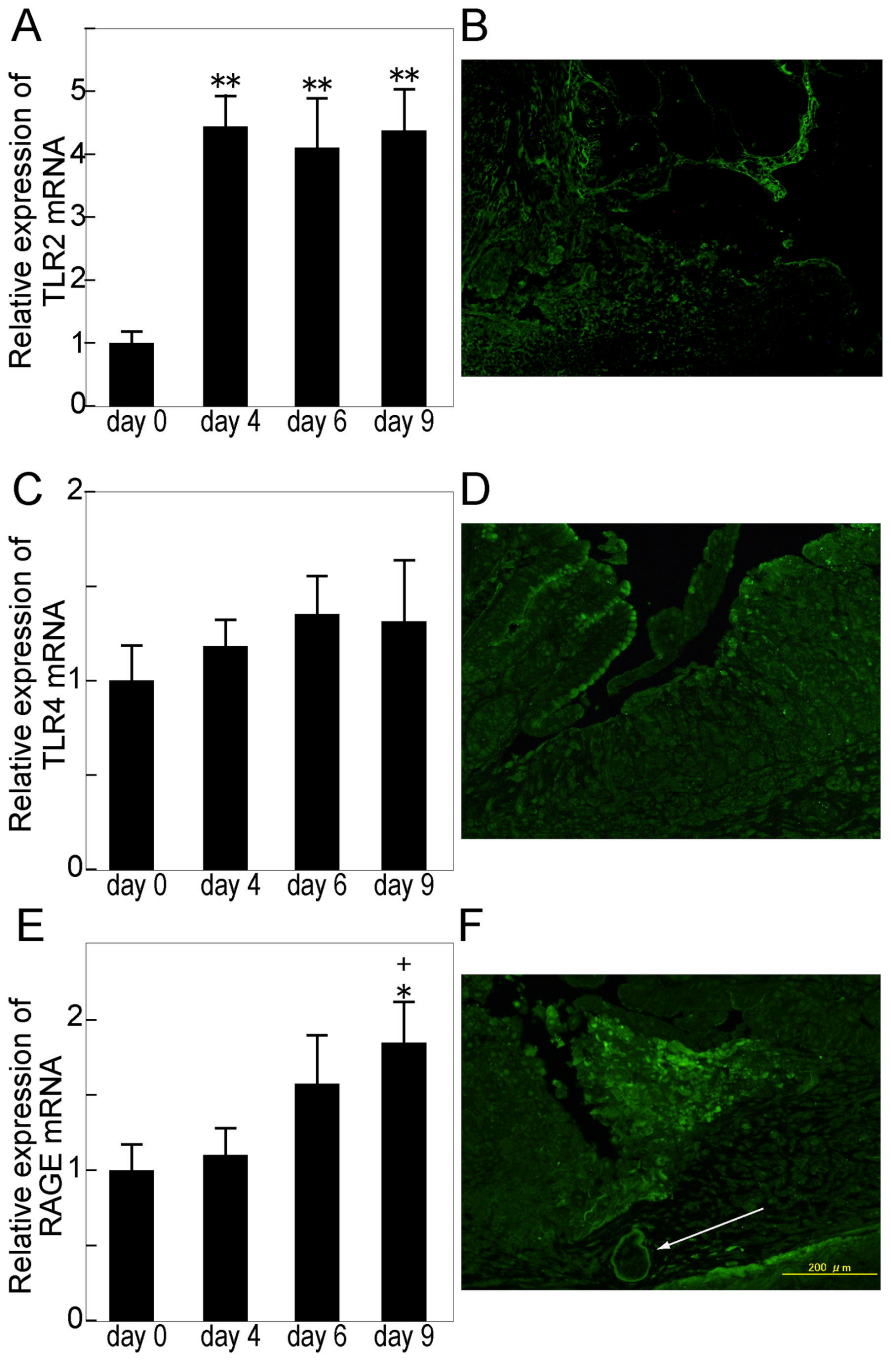
Expression of TLR2, TLR4 and RAGE during gastric ulcer healing. A, C, E: The expression of TLR2 (A), TLR4 (C) and RAGE (E) mRNA was determined by quantitative RT-PCR. mRNA levels are expressed as ratios, relative to the mean value for normal small gastric tissue. Each column represents the mean ± SEM. N = 6. **P < 0.01, *P < 0.05 vs. untreated control group. ^+^P < 0.05 vs. day 4 group. B, D, F: Immunohistochemical staining of TLR2 (B), TLR4 (D) and RAGE (F) in gastric ulcer tissue on day 6. TLR2 immunoreactivity was observed mainly in inflammatory cells (B). TLR4 immunoreactivity (D) was observed in the apical part of the epithelial lining at the ulcer edge and in some inflammatory cells in the ulcer bed. RAGE immunoreactivity (F) was observed mainly in inflammatory cells at the edge of ulcer beds and in the vascular endothelial cell membrane (arrow).

We next investigated mRNA expression and immunoreactivity of TLR2, TLR4 and RAGE in wild-type mice. A significant up-regulation of TLR2 mRNA was observed following the induction of an ulcer (Figure 6A). TLR2 immunoreactivity was observed mainly in inflammatory cells (Figure 6B). The expression of TLR4 mRNA was not affected by the induction of ulcer (Figure 6C). TLR4 immunoreactivity was observed in the apical part of the epithelial lining at the ulcer edge and in some inflammatory cells in the ulcer bed (Figure 6D).

A significant up-regulation of RAGE mRNA was observed on day 9 after the induction of ulcer (Figure 6E). RAGE immunoreactivity was observed mainly in inflammatory cells at the edge of the ulcer beds and in the vascular endothelial cell membrane (Figure 6F).

Staining of ulcerated tissue from TLR4 KO or RAGE KO mice with an anti-TLR4 antibody or anti-RAGE antibody, respectively, revealed no positive signals, confirming the specificity of these antibodies (data not shown). 

## Discussion

In this study, we demonstrated that exogenous HMGB1 delays gastric ulcer healing, while inducing TNFα expression and MPO activity. Conversely, immunoneutralization of HMGB1 or inhibiting the release of HMGB1 promotes ulcer healing while reducing TNFα expression and MPO activity. Additionally, TLR4 and RAGE deficiency promotes ulcer healing, and exogenous HMGB1 fails to delay ulcer healing in TLR4 KO and RAGE KO mice. These results suggest that HMGB1 is a complicating factor for gastric ulcer healing that acts through TLR4- and RAGE-dependent pathways. To our knowledge, this is the first report to clarify the role of HMGB1 in wound healing within the gastrointestinal tract. 

A few studies addressed the role of HMGB1 in wound healing in organs other than the gastrointestinal tract, but the results of these studies are controversial [37-40]. Consistent with our results, some studies demonstrated that HMGB1 has an inhibitory effect on wound healing [37,38]. Zhang et al. demonstrated that HMGB1 impairs incision wound healing by reducing reparative collagen deposition via RAGE [37]. Goova et al. demonstrated that blocking RAGE ligands such as AGE and HMGB1 by using a soluble form of receptor for AGE (sRAGE), accelerates ulcer healing and suppresses the levels of inflammatory cytokines [38]. Previous studies demonstrated that excessive inflammation impaired wound healing. For example, our previous study demonstrated that TNFα over-expression and excessive neutrophil infiltration are complicating factors in the formation and healing of gastric ulcer [41]. Furthermore, in another model of tissue repair, Goren et al. demonstrated that excessive neutrophil and macrophage infiltration with TNFα over-expression inhibits the healing of mouse skin injuries [42]. Thus, it is possible that HMGB1 delays gastric ulcer healing through TNFα-triggered inflammatory responses. 

In contrast, some in vitro studies suggested that HMGB1 and its receptors are essential for wound healing [39,40]. Staraino et al. demonstrated that HMGB1 accelerates the wound healing process and regeneration by enhancing the migration of skin fibroblasts and keratinocytes [39]. HMGB1 also promotes the wound healing of 3T3 fibroblasts by inducing cell proliferation and migration through the activation of the RAGE/extracellular signal-regulated kinase pathway [40]. Because these in vitro studies were performed in the absence of inflammatory cells, the differences in experimental methods may result in inconsistent conclusions on the role of HMGB1 in wound healing.

VEGF, a potent angiogenic growth factor, plays an important role in gastric ulcer healing [43,44]. Previous reports demonstrated that HMGB1 could induce the expression of VEGF in several tissues and animal models [45-47]. However, in this study, neither rHMGB1 nor anti-HMGB1 antibodies affected VEGF expression in ulcerous tissue. The reason for the disparity between our findings and those of earlier investigators is not clear, but it may be associated with the use of different organs and experimental models. 

There are 2 possible sources of extracellular HMGB1: passive release from necrotic cells [48] and active secretion from inflammatory cells [5]. In this study, HMGB1 immunoreactivity was observed in the cytoplasm as well as the nucleus, suggesting that necrotic cells are a source of HMGB1. Gastric HMGB1 mRNA levels increased during ulcer healing, suggesting that inflammatory cells produce and release HMGB1. Collectively, the elevation of serum HMGB1 resulted was due to passive release from injured cells at the gastric ulcer and active secretion from inflammatory cells. 

Serum HMGB1 levels increased following the induction of gastric ulcer but declined at the late phase of healing, although the expression of HMGB1 and TNFα remained high. These data raise the possibility of a systemic HMGB1 trapping system. To this end, the existence of an HMGB1 binding protein has been reported. Thrombomodulin, a cell-surface glycoprotein, is one example of an HMGB1 binding protein. Recombinant human soluble thrombomodulin inhibited the increase in plasma HMGB1 induced by lipopolysaccharide in a rat model [49] and bound to HMGB1 through its lectin domain [50] to prevent HMGB1 from interacting with other receptors [51]. In clinical applications, thrombomodulin is also useful in HMGB1-related diseases and conditions such as sepsis because of its anti-HMGB1 properties [52]. sRAGE, found in the circulation, is another example of an HMGB1 binding protein. sRAGE is the soluble form of RAGE; it acts as a decoy to prevent interaction between cell surface RAGE and its ligands, such HMGB1 [53]. In the present study, such trapping systems might also play a protective role in preventing the spread of inflammation, thereby promoting ulcer healing. 

It is known that many peptic ulcer patients are infected with *Helicobacter pylori*. Although in the present study we did not investigate the role of HMGB1 in gastric ulcer healing in mice infected with *H. pylori*, clinical and experimental studies suggest that the deleterious effect of HMGB1 on gastric ulcer healing would be more pronounced in patients with an *H. pylori* infection than in those without it. We previously showed that *H. pylori* infection increases neutrophil infiltration into ulcerated tissues in Mongolian gerbils [54]. Shimizu et al. also demonstrated that neutrophils and macrophages infiltrate ulcer margins to a higher degree in patients with *H. pylori* infection than in those without the infection [55]. A large amount of HMGB1 is, therefore, likely present in the ulcerated tissue infected with *H. pylori*, since it would be secreted by those inflammatory cells. Furthermore, Radin et al. reported that VacA, a major virulence factor of this organism, causes programmed necrosis of gastric epithelial cells and subsequent release of HMGB1 [56]. Thus, we expect that the deleterious of HMGB1 on ulcer healing would be more prominent in *H. pylori*-infected patients. 

TLR2, TLR4, and RAGE, which mediate proinflammatory responses, are commonly known HMGB1 receptors. Our results clearly showed that TLR4 and RAGE play crucial roles in gastric ulcer healing. This result is consistent with previous findings on the inflammatory responses induced by HMGB1. One of the most established models involving the interaction between HMGB1 and these receptors is I-R injury. In hepatic I-R injury, TLR4-deficient mice exhibited less liver I-R damage; the damage in TLR4-deficient mice was not affected by rHMGB1 or anti-HMGB1 antibody [26]. Moreover, blocking RAGE protected against hepatocellular death and necrosis in the hepatic I-R injury model [57]. In a model of cardiac I-R injury, Andrassy et al. showed that RAGE-deficient mice displayed only slight inflammation resulting from cardiac I-R injury; the inflammation was not affected by the induction of rHMGB1 [27]. These findings suggest that the TLR4-HMGB1 and RAGE-HMGB1 interactions play a crucial role in I-R injury, although it is necessary to consider the differences in each organ. The other established model is systemic inflammation, such as sepsis [5,8,58]. In an experimental model of intra-abdominal sepsis, Susa et al. demonstrated that the HMGB1-RAGE interaction was closely associated with sepsis-induced diaphragmatic dysfunction [58]. In an in vivo systemic inflammation model generated by injection of exogenous HMGB1, Zoelen et al. demonstrated that HMGB1 induces the release of cytokines, activation of coagulation, and neutrophil recruitment through TLR4 and RAGE [8]. Thus, TLR4 and RAGE play critical roles in pathogenesis mediated by the HMGB1-associated pathway, including the pathway in our ulcer healing model. 

Our results indicate that TLR2 has no relationship to gastric ulcer healing: ulcer healing in TLR2 KO mice resembled that in wild-type mice. Although in vitro studies using macrophage cell lines indicated that the TLR2-HMGB1 pathway induces inflammatory responses [6,9], in vivo effects of TLR2 in HMGB1-mediated pathologies have not been reported. Thus, based on previous findings [8] and our results, the HMGB1-TLR2 pathway may play a minor role in the repair and pathogenesis of tissue injuries and inflammation. 

In conclusion, we have shown that HMGB1 is a complicating factor in the healing process of gastric ulcer as well as in other pathological conditions. Moreover, we have shown that HMGB1 inhibits ulcer healing through a mechanism that involves TLR4, RAGE, and excessive inflammatory responses. Although proton pomp inhibitors are commonly prescribed for gastric ulcers, intractable ulcers still pose a clinical problem. Our present study supports a new concept for the treatment of intractable gastric ulcers, besides proton pomp inhibitors therapy. 
